# Fucoidan Inhibits the Growth of Hepatocellular Carcinoma Independent of Angiogenesis

**DOI:** 10.1155/2013/692549

**Published:** 2013-05-12

**Authors:** Cong Zhu, Rui Cao, Shuang-Xia Zhang, Ya-Nan Man, Xiong-Zhi Wu

**Affiliations:** ^1^Zhong-Shan-Men In-Patient Department, Tianjin Medical University Cancer Institute and Hospital, Huan-Hu-Xi Road, He-Xi District, Tianjin 300060, China; ^2^Department of Oncology, Shandong Provincial Chest Hospital, Jinan 250013, China

## Abstract

Some sulphated polysaccharides can bind bFGF but are unable to present bFGF to its high-affinity receptors. Fucoidan, a sulphated polysaccharide purified from brown algae, which has been used as an anticancer drug in traditional Chinese medicine for hundreds of years, exhibits a variety of anticancer effects, including the induction of the apoptosis and autophagy of cancer cells, the inhibition of the growth of cancer cells, the induction of angiogenesis, and the improvement of antitumour immunity. Our research shows that fucoidan dose not inhibit the expressions of VEGF, bFGF, IL-8, and heparanase in HCC cells and/or tumour tissues. Moreover, fucoidan exhibited low affinity for bFGF and could not block the binding of bFGF to heparan sulphated. Although fucoidan had no effect on angiogenesis and apoptosis *in vivo*, this drug significantly inhibited the tumour growth and the expression of PCNA. These results suggest that fucoidan exhibits an anticancer effect *in vivo* at least partly through inhibition of the proliferation of HCC cells, although it is unable to suppress the angiogenesis induced by HCC.

## 1. Introduction

Angiogenesis, which is the process of new blood vessel formation from preexisting vasculature, is crucial for malignant tumour growth [1, 2]. Proangiogenic molecules, such as basic fibroblast growth factor (bFGF), vascular endothelial growth factor (VEGF), and IL-8, play important roles in angiogenesis and tumour growth. Interestingly, bFGF, which is a heparin-binding growth factor, is commonly sequestered within heparan sulphate proteoglycans (HSPGs) in the basement membrane/extracellular matrix (ECM) in an inactive manner [3–5]. Heparanase specifically degrades the heparan sulphate chain of HSPGs to release active bFGF from sequestration in the ECM [6–8]. Once released, bFGF exerts its proangiogenic activity through binding to its specific FGF receptor (FGFR) located on the surface of endothelial cells (ECs) [9]. This process critically requires the involvement of the low-affinity receptor heparin/heparan sulphate (HS) side chains of HSPG to form the HS/FGF/FGFR ternary complex [10–12]. Compelling evidence indicates that a high level of bFGF is associated with tumour escape from antiangiogenic therapy [13]. Thus, targeting bFGF may be an efficient strategy for the suppression of angiogenesis and tumour growth [14].

Many recent studies have shown that sulphated polysaccharides are angiogenesis inhibitors. Sulphated polysaccharide can bind bFGF but is unable to present bFGF to its high-affinity receptors [15]. Moreover, sulphated polysaccharides can inhibit heparanase, which cleaves the heparan sulphate chains of heparan sulphate proteoglycans and causes the release of the growth factors sequestered by heparan sulphate chains [15]. The downregulation of the expression of heparanase inhibits the angiogenesis induced by hepatocellular carcinoma (HCC) cells both *in vitro* and *in vivo* [15]. PI-88, which is a complex mixture of highly sulphated oligosaccharides, has progressed to phase III clinical trials in postresection HCC, and this treatment represents the approach of developing heparin/HS-mimics to competitively interfere the formation of the HS/bFGF/FGFR complex [16–18]. GSPP, which is a novel sulphated polypeptide isolated from *Gekko swinhonis Guenther,* inhibits tumour angiogenesis by blocking bFGF production, release from ECM, and binding to its low-affinity receptor, heparin/heparan sulphate [19–23]. PVSP, which is a sulphated polysaccharide extracted from *Prunella vulgaris*, can influence the proliferation, apoptosis, and migration of endothelial cells, and this effect can be ascribed to the competitive ability of PVSP to inhibit the binding of bFGF to heparan sulphate [24].

*Sargassum* spp. (brown seaweed) has been used in traditional Chinese medicine (TCM) for nearly 2,000 years to treat a variety of diseases, including cancer [25]. Fucoidan, which is a sulphated polysaccharide purified from *Sargassum*, possesses a variety of anticancer effects, including the inhibition of the growth of HCC Huh7 cells and HepG2 cells, the induction of apoptosis and autophagy of human MCF-7 breast cancer cells, the Lewis lung carcinoma cells, melanoma B16 cells, and AGS human gastric adenocarcinoma cells, and the induction of the functional maturation of human monocyte-derived dendritic cells, T-cells, and natural killer cells [26–31]. Lv et al. reported that fucoidan inhibits both T98G and THP1 cell-induced angiogenesis [32]. However, the mechanisms of action through which fucoidan exhibits anticancer activity have not been fully elucidated. In this paper, we report that fucoidan inhibits tumour growth independently of angiogenesis. 

## 2. Methods

### 2.1. Reagents, Cell Lines, and Mice

Fucoidan was purchased from Ze Lang Nanjing Medical Technology Co., Ltd. (Nanjing, China). The HCC cell lines Bel-7402 and HepG2 were provided by Tianjin Medical University Cancer Research Institute [19, 21, 22]. The Bel-7402 and HepG2 cells were maintained in DMEM supplemented with 10% FBS. Female nu/nu nude mice were obtained from the Chinese Medical Academy of Science and used according to the National Institutes of Health guidelines for animal care. All surgeries were performed under sodium pentobarbital anaesthesia, and all efforts were made to minimise suffering. All *in vivo* studies were performed in the Centre Laboratory of Tianjin Medical University Cancer Institute, which is the authentic Animal Laboratory of the Committee on the Ethics of Tianjin Medical University Cancer Institute and Hospital.

### 2.2. Enzyme-Linked Immunosorbent Assay

The HepG2 cells were seeded at a density of 5 × 10^4^ cells/well in a 12-well plate and cultured for 24 hours. The medium was then replaced with fresh medium containing the indicated concentrations of fucoidan, and the cells were cultured for 72 hours. The culture supernatants were collected at 24, 48, and 72 hours. The levels of bFGF, VEGF, and IL-8 were determined by ELISA (R&D Systems) according to the manufacturer's instructions. The HepG2 cells were then lysed, and the levels of heparanase were detected using a heparan degrading enzyme assay kit (TAKARA).

### 2.3. Surface Plasmon Resonance (SPR) Assay

The kinetics and specificity of the binding reactions of recombinant human bFGF (PeproTech, Rocky Hill, NJ, USA) with heparin or fucoidan were analysed using the Biacore X100 surface plasmon resonance apparatus. Briefly, the first step is the immobilisation of recombinant human bFGF on a CM5 sensor chip (GE Healthcare, Piscataway, NJ, USA) using the amine coupling kit (Pharmacia Biosensor AB, Uppsala, Sweden) according to the procedure described by the manufacturer. PBS was used as the mobile phase at a flow rate of 10 *μ*L/min. The carboxymethyl dextran matrix of the sensor chip surface was first activated with an injection of 70 *μ*L of the N-ethyl-N-[(dimethylamino) propyl]carbodiimide (EDC):N-hydroxysuccinimide (NHS) reagent mixture. Then, 70 *μ*L of 100 *μ*g/mL bFGF in 10 mM NaOAc (pH 5.7) was injected and allowed to covalently couple to the sensor surface. Finally, the unreacted sites were blocked through the injection of 70 *μ*L of 1 M ethanolamine (pH 8.5). A response unit of ~6,580 RU was achieved after immobilisation. The second step was to determine the binding affinity of bFGF for fucoidan. This was performed by injecting 15 *μ*L of 10 mg/mL fucoidan in PBS buffer onto the chip in which bFGF was immobilised. To determine the binding affinity of bFGF for heparin, 15 *μ*L of heparin (10 mg/mL, Solarbio Biology) was injected into buffer and flowed onto the chip in which bFGF was immobilised. To evaluate the effect of fucoidan on the binding of heparin to bFGF, fucoidan was added and bounded to the bFGF immobilised in the chip, and then 10 mg/mL heparin was added. To correct for nonspecific binding and bulk refractive index changes, a blank channel (FC2) without heparin and fucoidan was employed as a control for each experiment. All of the binding interactions were recorded in real time, and the blank channel readings were subtracted from the results before analysis. The changes in the mass due to the binding response were recorded as resonance units (RU). All of the binding experiments were performed at 25°C with a constant flow rate of 10 *μ*L/min PBS. The sensor chip surface was regenerated between the experiments through the injection of 10 *μ*L of 15 mM HCl plus 0.5 M NaCl and then switched to PBS. For the direct assessment of the dissociation constants, the association phase was allowed to proceed to equilibrium. The binding kinetics and the stoichiometry were determined by surface plasmon resonance using the Biacore software.

### 2.4. Solid-Phase Heparan Sulphate-bFGF Binding Assay

The heparan degrading enzyme assay kit (Takara, Otsu, Japan) was applied to observe the interaction between bFGF and a mixture of heparan sulphate/fucoidan, but the protocol was modified. Briefly, 50 *μ*L of 10 mg/mL fucoidan and 50 *μ*L of biotinylated heparan sulphate were mixed for 1 minute. The control group received 50 *μ*L of biotinylated heparan sulphate and 50 *μ*L of PBS. Subsequently, the mixture was transferred into CBD-FGF immobilised in a 96-well microtitre plate and incubated at 37°C for 15 minutes. After discarding the reaction solution and washing, 100 *μ*L of avidin POD conjugate was added to each well and incubated at 37°C for 30 minutes. Then, 100 *μ*L of the POD substrates were added to each well, and the colour was allowed to develop at room temperature for 15 minutes. Then, 100 *μ*L of the stop solution was added to each well. After the zero adjustment of the microplate reader using distilled water, the absorbance of each well was measured at 450 nm.

### 2.5. Tumour Xenograft Assay

Female nu/nu nude mice, aged from 4 to 6 weeks and weighing approximately 18–20 g, were injected subcutaneously with Bel-7402 cells (0.1 mL, 8 × 10^6^). The mice were allocated into three groups according to the treatments: (1) normal saline (control group), (2) 20 mg/kg fucoidan, and (3) 200 mg/kg fucoidan. The administration of the drug began the day after the inoculation, and each animal received the drug once a day over a period of 25 days through a cavitas abdominalis injection. Each tumour was measured every three days in two dimensions (length and width) using callipers, and the tumour volume (mm^3^) was calculated (*V* = length × width^2^/2). On the 25th day, animals were weighted and then sacrificed. The implanted tumours were excised and weighted. 

### 2.6. Immunohistochemical Staining and Vessel Density Measurements

Sections (4-*μ*m thick) from formalin-fixed paraffin-embedded tumour xenografts were prepared. After deparaffinisation, primary rat monoclonal anti-mouse antibodies against CD34 (1 : 10 dilution, Abcam), PCNA (1 : 100 dilution, Abcam), and bFGF (1 : 20 dilution, Santa Cruz Biotechnology) were used to perform the immunohistochemical analysis according to the manufacturer's instructions. To evaluate the microvessel density, the tumour tissue sections were examined at low magnification to identify the areas with the highest density of CD34-positive vessels. The numbers of microvessels in the 400x fields were counted. To quantify the PCNA expression, the sections were photographed with a digital camera at 400x magnification. The quantification of the PCNA density was performed using the Image-Pro Plus image analysis software. For each section, the integral optical density of every visual field was calculated. 

### 2.7. TUNEL Staining

Sections (4-*μ*m thick) from formalin-fixed paraffin-embedded tumour xenografts were prepared. The apoptotic cells were detected using commercially available TUNEL (terminal deoxynucleotidyl transferase dUTP nick end labelling) assay kits (Promega) according to the instructions provided by the manufacturer. The sections were photographed with a confocal microscope at a magnification of 40x.

### 2.8. Statistical Analysis

The statistical significance was analysed by one-way ANOVA using the SPSS 16.0 software. Differences with a *P* value less than 0.05 were considered statistically significant. 

## 3. Results

### 3.1. Effects of Fucoidan on the Expression of bFGF, VEGF, IL-8, and Heparanase *In Vitro*

We observed the expression of the proangiogenic molecules bFGF, VEGF, and IL-8 in HepG2 cells. Fucoidan had no effect on the levels of bFGF. Moreover, fucoidan had no effect on the levels of VEGF and IL-8 (Table 1). Because heparanase enzymatically cleaves HS glycosaminoglycan chains from proteoglycans to release the active form of bFGF from the ECM, we attempted to assess the level of heparanase in the hepatoma cells after treatment with fucoidan. However, fucoidan had no effect on the levels of heparanase (Table 1). 

### 3.2. Effects of Fucoidan on the Binding between bFGF and Heparin/HS

**Figure 1:**
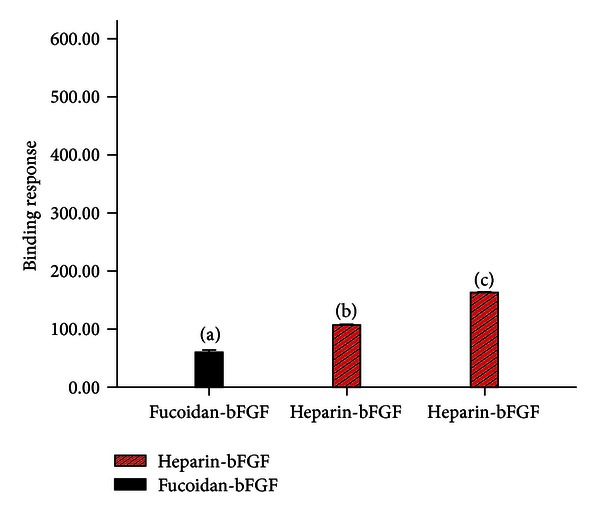
Effects of fucoidan on the binding between bFGF and heparin. (a) Interaction of fucoidan-bFGF. (b) Interaction between fucoidan, heparin, and bFGF. (c) Interaction between heparin and bFGF.

We tested the hypothesis that fucoidan may directly interact with bFGF through the SRP assay. The responses of fucoidan-bFGF and heparin-bFGF are 60.14 ± 4.13 RU and 163.24 ± 0.2 RU, respectively. After treatment with fucoidan, the response of heparin-bFGF was found to be 107.06 ± 0.23 RU (Figure 1). Moreover, a solid-phase combined assay was applied to ensure competitive binding between bFGF-heparan sulphate and bFGF-fucoidan. The binding ability of HS to bFGF did not change when HS was mixed with fucoidan prior to their interaction with bFGF. 

### 3.3. Effects of Fucoidan on the Expression Levels of bFGF and VEGF and Angiogenesis *In Vivo*

**Figure 2:**
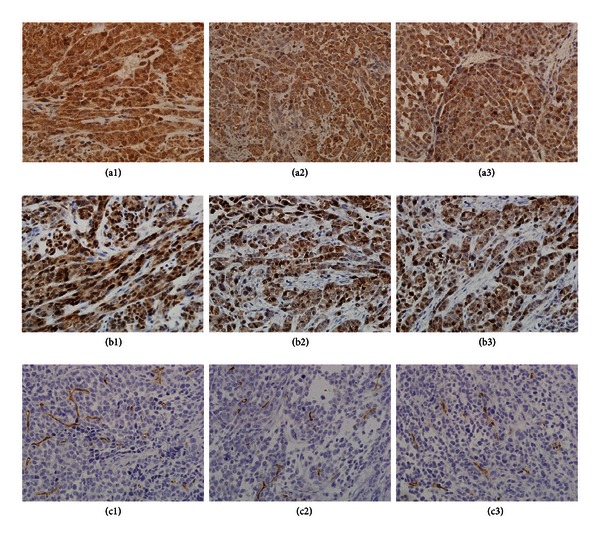
Effects of fucoidan on bFGF, VEGF, and angiogenesis *in vivo*. (a) Expression of VEGF in tumour tissue. (b) Expression of bFGF in tumour tissue. (c) Tumour tissue stained against CD34. (a), (b), and (c): 1, control; 2, 20 mg/kg fucoidan; 3, 200 mg/kg fucoidan.

Fucoidan has no effects on the expression of bFGF and VEGF in tumour tissue. Considering the hypervascular nature of HCC, we then evaluated the microvascular density (MVD) in tumour sections and found that fucoidan has no effect of the MVD in tumours compared with the control (Figure 2).

### 3.4. Effects of Fucoidan on Tumour Growth *In Vivo*

To determine the efficacy of fucoidan *in vivo*, Bel-7402 cells were implanted into the back of female nu/nu nude mice, and the growth of tumours was monitored at 3-day intervals. Fucoidan at a concentration of 200 mg/kg was capable of significantly decreasing the tumour volume (Table 2). The tumour weight in the groups treated with 20 mg/kg and 200 mg/kg fucoidan was reduced by 11.97% and 36.16%, respectively (Table 2). To evaluate the adverse effects of the drug, we measured the growth of mice (weight) and found that there was no difference between the control and the fucoidan-treated groups (Table 2).

### 3.5. Effects of Fucoidan on the Expression of PCNA *In Vivo*

**Figure 3:**
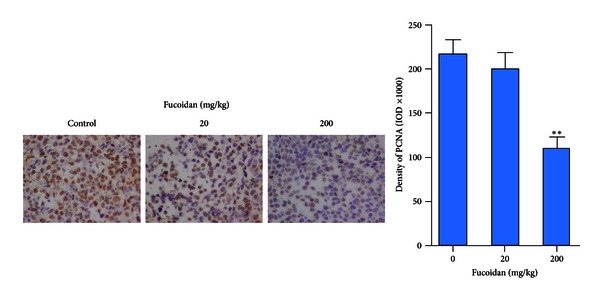
Effect of fucoidan on PCNA *in vivo*. Left: images of the PCNA-positive areas (400x magnification). Right: quantification of PCNA-positive areas. **P < 0.01 compared with control. IOD: integral optical density.

To quantify the PCNA expression, the sections were photographed with a digital camera at 400x magnification, and the integral optical density of PCNA in each section was analysed using the Image-Pro Plus image analysis software. We found that the expression of PCNA in the tumour tissues decreased significantly after treatment with 200 mg/kg fucoidan (Figure 3).

### 3.6. Effects of Fucoidan on Apoptosis *In Vivo*

**Figure 4:**
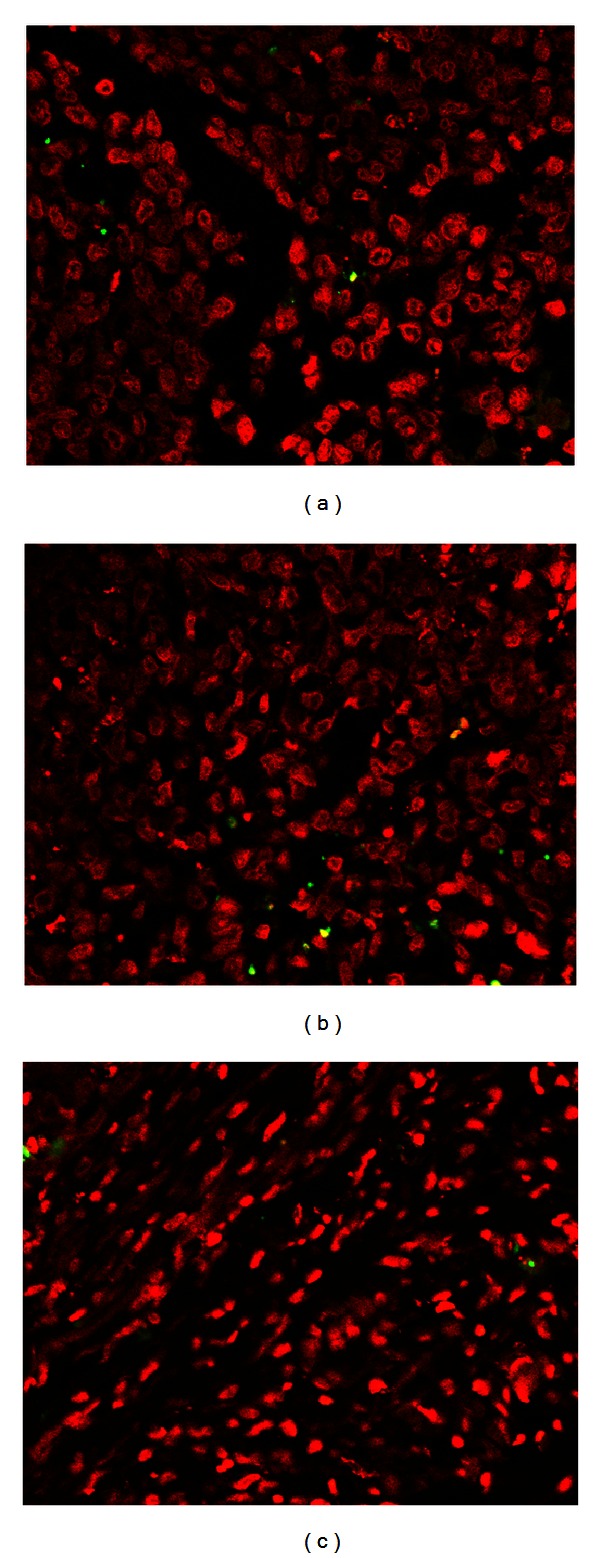
Effects of fucoidan on apoptosis *in vivo*. (a) Control, (b) 20 mg/kg fucoidan, and (c) 200 mg/kg fucoidan.

Through TUNEL staining, all of the cells were stained with a red fluorescence. Only the apoptotic cells exhibited a green fluorescence. The TUNEL detection showed that fucoidan could not induce apoptosis in tumour tissue. In all of the groups, there were very few apoptotic cells in the tumour tissue (Figure 4).

## 4. Discussion

Fucoidan, a sulphated polysaccharide purified from brown algae, which has been used as an anticancer drug in traditional Chinese medicine for hundreds of years, possesses a variety of anticancer effects, including the inhibition of the growth of HCC Huh7 cells and HepG2 cells, the induction of apoptosis and autophagy of human MCF-7 breast cancer cells, the Lewis lung carcinoma cells, melanoma B16 cells, and AGS human gastric adenocarcinoma cells, and the induction of a functional maturation of human monocyte-derived dendritic cells, T-cells, and natural killer cells [26–31]. Lv et al. reported that fucoidan inhibited both T98G and THP1 cell-induced angiogenesis [32]. Proangiogenic molecules, such as bFGF, VEGF, and IL-8, play important roles in angiogenesis. Interestingly, we found that fucoidan could not inhibit the expression of VEGF, bFGF, and IL-8 in HCC cells. Similar results were observed in tumour tissues.

The proangiogenic activity of bFGF, which is a heparin-binding growth factor, can be inhibited by different strategies, such as the reduction of bFGF production by tumour cells, the modulation of the release of active bFGF from the ECM, and the modification of the FGF-FGFR recognition [33, 34]. Recently, many research studies have shown that sulphated polysaccharides, such as PI-88, GSPP, which is a novel sulphated polypeptide isolated from *Gekko swinhonis Guenther*, and PVSP, which is a sulphated polysaccharide extracted from *Prunella vulgaris*, can bind bFGF but are unable to present bFGF to its high-affinity receptors [15]. Moreover, sulphated polysaccharides can inhibit heparanase, which cleaves the heparan sulphate chains of heparan sulphate proteoglycans and causes the release of the growth factors sequestered by the heparan sulphate chains [15]. In this study, we evaluated the effects of fucoidan on bFGF through different mechanisms. Interestingly, we found that fucoidan could not inhibit the expression of bFGF in HCC cells. Moreover, fucoidan could not suppress the heparanase-stimulated release of bFGF from the ECM.

An SPR assay was conducted to determine whether fucoidan could interact with bFGF directly. In this assay, fucoidan was added to a chip in which bFGF was immobilised prior to the addition of heparin. The results showed that fucoidan exhibited low affinity with bFGF. Heparin is the low-affinity receptor of bFGF. The responses of fucoidan-bFGF and heparin-bFGF were found to be 60.14 ± 4.13 RU and 163.24 ± 0.25 RU, respectively. After treatment with fucoidan, the response of heparin-bFGF was measured to be 107.06 ± 0.23 RU. Our previous research showed that the responses of GSPP-bFGF and PVSP-bFGF are 592.20 ± 0.10 RU and 691.26 ± 0.22 RU, respectively [19, 24]. A solid-phase combined assay was conducted to test the hypothesis that fucoidan could bind to bFGF in competition with HS. In this experiment, fucoidan and HS were mixed prior to their addition to a plate that was precoated with bFGF. Interestingly, it was found that fucoidan could not block the binding of heparan sulphate to bFGF.

To determine the efficacy of fucoidan *in vivo*, an HCC xenograft mouse model was used. Fucoidan significantly suppressed the tumour volume and the tumour weight. The tumour weight in the groups treated with 20 mg/kg and 200 mg/kg fucoidan was reduced by 11.97% and 36.16%, respectively. To evaluate the adverse effects of fucoidan, we measured the growth of mice (weight) and found that there was no difference between the control and the fucoidan-treated groups. 

We further investigated the effects of fucoidan on the angiogenesis induced by HCC *in vivo*. Treatment with 20 mg/kg fucoidan and 200 mg/kg fucoidan could not suppress the MVD in tumour tissues compared with the control mice. Moreover, through TUNEL staining, we found that fucoidan had no effect on the apoptosis of HCC *in vivo*.

Fucoidan inhibited the growth of hepatoma Huh7 cells and HepG2 cells in a dose-dependent manner* in vitro* [28]. Proliferating cell nuclear antigen, which is commonly known as PCNA, is a protein that acts as a factor for DNA polymerase *δ* in eukaryotic cells. This protein achieves this processivity by encircling the DNA, thus creating a topological link to the genome. Hepatoma cells exhibit vigorous proliferation activity, and PCNA can be used as an indicator of the state of cell proliferation. The expression of PCNA in tumour tissues was improved, and fucoidan could inhibit the expression of PCNA. Thus, fucoidan exhibits its anticancer effect at least partly through the inhibition of the proliferation of cancer cells *in vivo*. Chemokine ligand 12 (CXCL12; stromal cell-derived factor 1) is a homeostatic chemokine that signals through chemokine receptor 4 (CXCR4), which plays an important role in the proliferation of hepatoma cells [35, 36]. The effect of CXCL12 is inhibited by heparin, which is a sulphated polysaccharide [37]. Mavier et al. [38] showed that fucoidan binds to CXCL12 and blocks its biological effects in an AAF/PH model. Nagamine et al. reported that fucoidan exhibits antitumor activity toward Huh7 cells *in vitro* through the downregulation of CXCL12 expression [28]. Thus, it is of interest to determine whether fucoidan inhibits the growth of HCC *in vivo* through blocking the biological effects of the CXCL12/CXCR4 axis. 

## 5. Conclusions

Taken together, the results show that fucoidan exhibits antitumour activity *in vivo*. Fucoidan exhibits this anticancer effect at least partly through inhibition of the proliferation of HCC cells. However, fucoidan does not suppress the angiogenesis induced by HCC.

## Figures and Tables

**Table 1:** Effects of fucoidan on the expression of bFGF, VEGF, IL-8, and heparanase.

Group	bFGF (pg/mL)	VEGF (pg/mL)	IL-8 (pg/mL)	Heparanase (U/mL)
Control	1018.78 ± 75.15	26.168 ± 3.431	80.41 ± 19.72	0.0673 ± 0.0134
10 *µ*g/mL	1148.89 ± 122.76	23.505 ± 3.452	66.87 ± 4.48	0.1004 ± 0.0288
100 *µ*g/mL	1152.11 ± 80.30	22.998 ± 4.752	74.21 ± 2.97	0.0593 ± 0.0026
200 *µ*g/mL	1136.56 ± 84.74	30.861 ± 6.068	54.46 ± 1.29	0.0849 ± 0.0185

**Table 2:** Effects of fucoidan on the growth of mice (weight), the tumour volume, and the tumour weight.

Group	Growth of mice (weight, g)	Tumour volume (mm^3^)	Tumour weight (g)
200 mg/kg fucoidan	5.58 ± 1.18	301.70 ± 149.37^▲^	0.40 ± 0.15^▲^
20 mg/kg fucoidan	5.76 ± 1.34	416.03 ± 198.32	0.58 ± 0.20
Control	5.81 ± 1.52	472.62 ± 211.89	0.59 ± 0.20

^▲^ *P* < 0.05 compared with control.
